# Auricular Transcutaneous Vagus Nerve Stimulation Acutely Modulates Brain Connectivity in Mice

**DOI:** 10.3389/fncel.2022.856855

**Published:** 2022-04-25

**Authors:** Cecilia Brambilla-Pisoni, Emma Muñoz-Moreno, Ianire Gallego-Amaro, Rafael Maldonado, Antoni Ivorra, Guadalupe Soria, Andrés Ozaita

**Affiliations:** ^1^Laboratory of Neuropharmacology, Department of Medicine and Life Sciences, Universitat Pompeu Fabra, Barcelona, Spain; ^2^Experimental 7T MRI Unit, Magnetic Resonance Imaging Core Facility, Institut d'Investigacions Biomediques August Pi i Sunyer, Barcelona, Spain; ^3^Institut Hospital del Mar d'Investigacions Mèdiques (IMIM), Barcelona, Spain; ^4^Department of Information and Communication Technologies, Universitat Pompeu Fabra, Barcelona, Spain; ^5^Serra Húnter Fellow Programme, Universitat Pompeu Fabra, Barcelona, Spain; ^6^Laboratory of Surgical Neuroanatomy, Faculty of Medicine and Health Sciences, Institute of Neurosciences, University of Barcelona, Barcelona, Spain

**Keywords:** auricular transcutaneous vagus nerve stimulation, memory persistence, brain connectivity, electrostimulation, c-Fos functional networks

## Abstract

Brain electrical stimulation techniques take advantage of the intrinsic plasticity of the nervous system, opening a wide range of therapeutic applications. Vagus nerve stimulation (VNS) is an approved adjuvant for drug-resistant epilepsy and depression. Its non-invasive form, auricular transcutaneous VNS (atVNS), is under investigation for applications, including cognitive improvement. We aimed to study the effects of atVNS on brain connectivity, under conditions that improved memory persistence in CD-1 male mice. Acute atVNS in the *cymba conchae* of the left ear was performed using a standard stimulation protocol under light isoflurane anesthesia, immediately or 3 h after the training/familiarization phase of the novel object-recognition memory test (NORT). Another cohort of mice was used for bilateral c-Fos analysis after atVNS administration. Spearman correlation of c-Fos density between each pair of the thirty brain regions analyzed allowed obtaining the network of significant functional connections in stimulated and non-stimulated control brains. NORT performance was enhanced when atVNS was delivered just after, but not 3 h after, the familiarization phase of the task. No alterations in c-Fos density were associated with electrostimulation, but a significant effect of atVNS was observed on c-Fos-based functional connectivity. atVNS induced a clear reorganization of the network, increasing the inter-hemisphere connections and the connectivity of locus coeruleus. Our results provide new insights into the effects of atVNS on memory performance and brain connectivity extending our knowledge of the biological mechanisms of bioelectronics in medicine.

## Introduction

Nowadays, brain stimulation devices have gained significant interest in the scientific community and have received European Medicine Agency (EMA) and US Food and Drug Administration (FDA) approvals for different therapeutic purposes. An approach to achieve brain stimulation is through vagus nerve afferents. Indeed, vagus nerve stimulation (VNS) has become an interesting strategy to help handle drug-resistant epilepsy and depression (Yuan and Silberstein, 2015). In this context, transcutaneous VNS (tVNS), given its non-invasiveness, has received substantial attention. tVNS can be applied to different locations, such as in the neck (Brock et al., 2017) or in the *cymba conchae* of the external ear (Peuker and Filler, 2002), and, similar to the invasive form of VNS, has already been approved as an adjuvant in the clinical applications, such as for the treatment of drug-resistant epilepsy and depression (Hein et al., 2013; Bauer et al., 2016). Surprisingly, the direct effect of tVNS over brain function is far from understood. Renewed attention to this electrostimulation technique derives from its neuromodulatory effect on cognitive processes. The vagus nerve afferent fibers in the brainstem end in the nucleus of the solitary tract (NTS) (Ruffoli et al., 2011), a relevant relay area for visceral information. From there, afferent information is distributed to many brain regions, including the *locus coeruleus* (LC) (Grimonprez et al., 2015). The LC provides a widespread innervation to the hippocampus, amygdala, and prefrontal cortex, among other regions (Ruffoli et al., 2011). In this regard, it has been postulated that the LC could regulate cognition through the release of norepinephrine and dopamine in memory processing areas (Ruffoli et al., 2011; Duszkiewicz et al., 2019), mimicking those physiological processes involved in attention-driven cognition (Mello-Carpes and Izquierdo, 2013; Mather and Harley, 2016; Zerbi et al., 2019), but whether such interaction results in the modulation of brain networks is not completely understood. Among those brain networks supporting brain activity, the default mode network (DMN), a brain network predominantly active when the brain is not engaged in an attention-driven task (Raichle et al., 2001; Stafford et al., 2014), has received much consideration due to the reproducibility in its detection in the clinical and preclinical settings (Stafford et al., 2014). The DMN is therefore disengaged during periods of active brain function, as during attention-associated periods, and it has been described to be modulated by tVNS in patients with mild or moderate depressive symptoms (Fang et al., 2016). Focusing on the cognitive functions associated with memory, our group already reported that auricular tVNS (atVNS) enhanced novel object-recognition (NOR) memory persistence in *naïve* CD-1 mice (Vázquez Oliver et al., 2020). The brain mechanisms recruited by atVNS to enhance memory performance have not been described, so we further explored the cellular outcome of atVNS under conditions that potentiate object-recognition memory persistence. First, we assessed whether a critical time window for atVNS efficacy may limit its effect on favoring object-recognition memory. Second, we analyzed the expression of an immediate early gene c-Fos as an approach to study brain activity in discrete brain regions (Guzowski et al., 2005). In line with the current view proposing that specific cognitive functions are supported by a network of functionally connected brain regions, rather than isolated areas, together with the region-specific analysis of c-Fos, we also evaluated the c-Fos-based functional network (Vetere et al., 2017). We focused our analysis on areas of the brainstem, hippocampus, amygdala, thalamus, and frontal/dorsal cortex, some of which are components of the DMN. Starting from these c-Fos data, we analyzed and estimated the functional connectivity network based on c-Fos density for both stimulated and non-stimulated brains to find significant changes in network connectivity patterns in atVNS condition.

## Materials and Methods

### Animals

Young adult male CD-1 mice (10 weeks old) were purchased from the Charles River Laboratories (France). All the experimental mice were bred at the Barcelona Biomedical Research Park (PRBB) Animal Facility. All animal procedures were conducted in accordance with the standard ethical guidelines (European Communities Directive 2010/63/EU). Mice were housed in a temperature-controlled (21 ± 1°C) and humidity-controlled (55 ± 10%) environment. Lighting was maintained at 12 h' cycles (on at 8 a.m. and off at 8 p.m.). Food and water were available *ad libitum*. Mice were handled for 1 week before starting the experiment and were randomly distributed among experimental groups. All the procedures were performed by experimenters blind to the experimental conditions.

### Experimental Design—Behavioral and Electrostimulation Procedures

The electrostimulation was performed at the time points indicated later after the familiarization/training phase in the novel object-recognition test (NORT), following a similar approach to that described earlier (Vázquez Oliver et al., 2020). Briefly, on the habituation phase performed on day 1, mice were habituated to an empty V-shape maze (V-maze) for 9 min. The next day, on the training phase performed on day 2, mice were presented in the V-maze to two identical objects for 9 min, each object at the end of the maze corridors. Immediately after the familiarization phase [atVNS (0 h), *n* = 11] or 3 h after the familiarization phase [atVNS (3 h), *n* = 12] mice were anesthetized with isoflurane (1.5%) in 0.8 L/min O_2_ during 30 min, and subjected or not to atVNS. Normothermic conditions were maintained during anesthesia with a heating pad. For atVNS (0 h) and atVNS (3 h) conditions, a newly custom-designed bipolar electrode (described in Vázquez Oliver et al., 2020) was placed in the *cymbae concha* of the left ear. Rectangular biphasic pulses were delivered with a Beurer EM49 stimulator (Beurer, Germany). The stimulation parameters were: 1 mA, 20 Hz, 30 s ON and 5 min OFF, the total length of 30 min, with a 320 μs pulse width. For the No stimulation condition (*n* = 12), mice were anesthetized for 30 min immediately after the NORT familiarization, but no electrical stimulation was delivered. Forty eight h after the NORT familiarization phase and the atVNS or No stimulation procedures, mice were tested for 9 min in the V-maze, substituting one of the familiar objects for a new one, to assess memory performance.

### Tissue Preparation for Immunofluorescence

In another batch of animals, the exact same NORT + atVNS or NORT + No stimulation protocols described earlier were followed, returning the mice to their home-cage afterward. atVNS nomenclature corresponds to atVNS (0 h) when not otherwise specified. Ninety minutes following the completion of the NORT + atVNS [similar to atVNS (0 h) condition, *n* = 8] or the NORT + No stimulation (No stimulation condition, *n* = 8), mice were deeply anesthetized by intraperitoneal injection (0.2 ml/10 g of the body weight) of a mixture of ketamine (100 mg/kg) and xylazine (20 mg/kg) prior to intracardiac perfusion with 4% paraformaldehyde in 0.1 M Na_2_HPO_4_/0.1 M NaH_2_PO_4_ buffer (PB), pH 7.5, delivered with a peristaltic pump at 19 ml/min flow for 3 min. Subsequently, the brain was extracted and post-fixed in the same fixative solution for 24 h and transferred to a solution of 30% sucrose in PB overnight at 4°C. After postfixation, the brains were marked in the right hemisphere to preserve laterality in the subsequent measures. Coronal sections of 30 μm were obtained on a freezing microtome and stored in a solution of 5% sucrose at 4°C until used.

### Immunofluorescence

Sections from the No stimulation and the atVNS groups were processed in parallel for immunofluorescence. Briefly, free-floating brain slices were rinsed in PB, blocked in a solution containing 3% normal goat serum (GS) (S-1000-20, Vector Laboratories Incorporation, California, USA) and 0.3% Triton X-100 (T) in PB (GS-T-PB) at room temperature for 2 h, and incubated overnight in the same solution with the primary antibody to c-Fos (sc-7202, 1:1,000, rabbit, Santa Cruz Biotechnology) and, only for LC slices, with tyrosine hydroxylase (T1299, 1:1,000, mouse, Sigma-Aldrich) at 4°C. The next day, after 3 rinses in PB, sections were incubated at room temperature with the secondary antibody AlexaFluor-555 goat anti-rabbit (ab150078, 1:1,000, Abcam) and, only for LC slices, with AlexaFluor-488 goat anti-mouse (115-545-003, 1:1,000, Jackson ImmunoResearch Laboratories Incorporation) for 2 h. After incubation, sections were rinsed and mounted immediately after onto glass slides coated with gelatin in Fluoromont-G with 4',6-diamidino-2-phenylindole (DAPI) (00-4959-52, Invitrogen, Thermo Fisher Scientific, Massachusetts, USA) as counterstaining.

### c-Fos Quantification

c-Fos density was analyzed in thirty brain regions (fifteen per hemisphere), taking into account brain laterality. Analyzed brain regions included (from frontal to caudal): cingulate cortex (Cg), prelimbic cortex (PrL), infralimbic cortex (IL) (coordinates relative to Bregma: 1.94–1.54 mm), dentate gyrus (DG), CA1 and CA3 areas of the hippocampus (from Bregma: −1.46 to −1.82 mm), basolateral amygdala (BLA), lateral amygdala (LA) and central amygdala (CeA) (from Bregma: −1.46 to −1.82 mm), paraventricular nucleus of the thalamus (PVT) (from Bregma: −1.46 to −1.82 mm), anterior and posterior retrosplenial cortex (RSP, pRSP) (from Bregma: −1.46 to −2.92 mm), *locus coeruleus* (LC) (from Bregma: −5.34 to −5.68 mm), the nucleus of the solitary tract (NTS), and dorsal vagal nucleus (DMX) (from Bregma: −7.32 to −7.64) (Supplementary Figure 1). The immunostained brain sections were analyzed with a 10X objective using a Leica DMR microscope (DM6000B, Leica Microsystems, Wetzlar, Germany) equipped with a digital camera Leica DFX 3000FX (Leica Microsystems). The borders of all the regions were defined manually according to the mouse brain atlas (Paxinos and Franklin, 2019). For prelimbic, infralimbic, and cingulate cortexes analysis, a 430-μm-sided square region of interest (ROI) was delimited for quantification. For amygdala and dorsal hippocampus analysis, the DAPI signal was used for the delimitation of the areas in each image for quantification. For the LC, the tyrosine hydroxylase signal was used for the delimitation of the area in each image for quantification. The images were processed using ImageJ software (Rasband, 1997-2018). c-Fos-positive cells in each brain area were quantified manually using the cell counter plugin of ImageJ software. The average number of c-Fos-positive cells on four determinations for each brain area on each hemisphere were calculated for each mouse. The c-Fos density for each region was quantified by dividing the number of c-Fos-positive cells to the area considered for each region (c-Fos+/mm^2^) (see Supplementary Figure 1 for representative examples).

### Generation of Functional Connectivity Network

The functional network was estimated for each condition based on the correlation between regional c-Fos density, considering that a functional connection exists between the two regions if their activity covaries (Park and Friston, 2013; Vetere et al., 2017). Therefore, within each experimental group (No stimulation and atVNS), the pair-wise Spearman's correlation coefficient between each pair of regional c-Fos density was calculated. In this way, a correlation matrix was obtained from each condition representing the correlation coefficients between all thirty brain regions analyzed, taking into account the brain laterality. By considering only significant correlations (*p* < 0.05), both the positive and negative, we obtained the condition-related functional network for atVNS and No stimulation protocols. These networks were represented by circos plots, using a custom R-code (R version 4.0.4) (R Core Team, 2020). Finally, we computed the z-Fisher transform of significant positive correlation coefficients, as a measure of connectivity strength between nodes (z-score), for both conditions and displayed them by Kamada-Kawai graphs using NetworkX graph python package (NetworkX version 2.5.1) (Hagberg et al., 2008) to visualize network organization.

### Network Analysis

First, the total functional connectivity strengths for all the possible connections were compared between atVNS and No stimulation conditions, considering z-score. Likewise, for the LC region, we compared its connectivity strength with all the other evaluated regions between atVNS and non-stimulated networks.

To have a global characterization of the condition-related functional connectivity, we also computed graph metrics on the network of significant positive connectivity strengths using Brain Connectivity Toolbox (BCT) (Rubinov and Sporns, 2010). In particular, global efficiency, average clustering, average strength, and average degree of the network were estimated. In addition, regional network metrics such as nodal strength and nodal degree coefficients were also computed. To compare network organization and the relevance of each region in the functional network, regional metrics were normalized to the maximum in the network and ordered from higher to lower value to identify network hubs (Wheeler et al., 2013).

### Statistical Analysis

Data were analyzed with STATISTICA (StatSoft) software using the one-way ANOVA for multiple comparisons of parametric variables. The Kruskal–Wallis test was used for non-parametric variables. Subsequent *post-hoc* analysis (Newman–Keuls test) was used when required to reveal a significant interaction between factors. The artwork was designed using GraphPad Prism version 7. Comparisons were considered statistically significant when *p* < 0.05. Data are represented as mean ± SEM.

## Results

### Object-Recognition Memory Enhancement by Acute Auricular Transcutaneous Vagus Nerve Stimulation Depends on the Time of Administration

**Figure 1:**
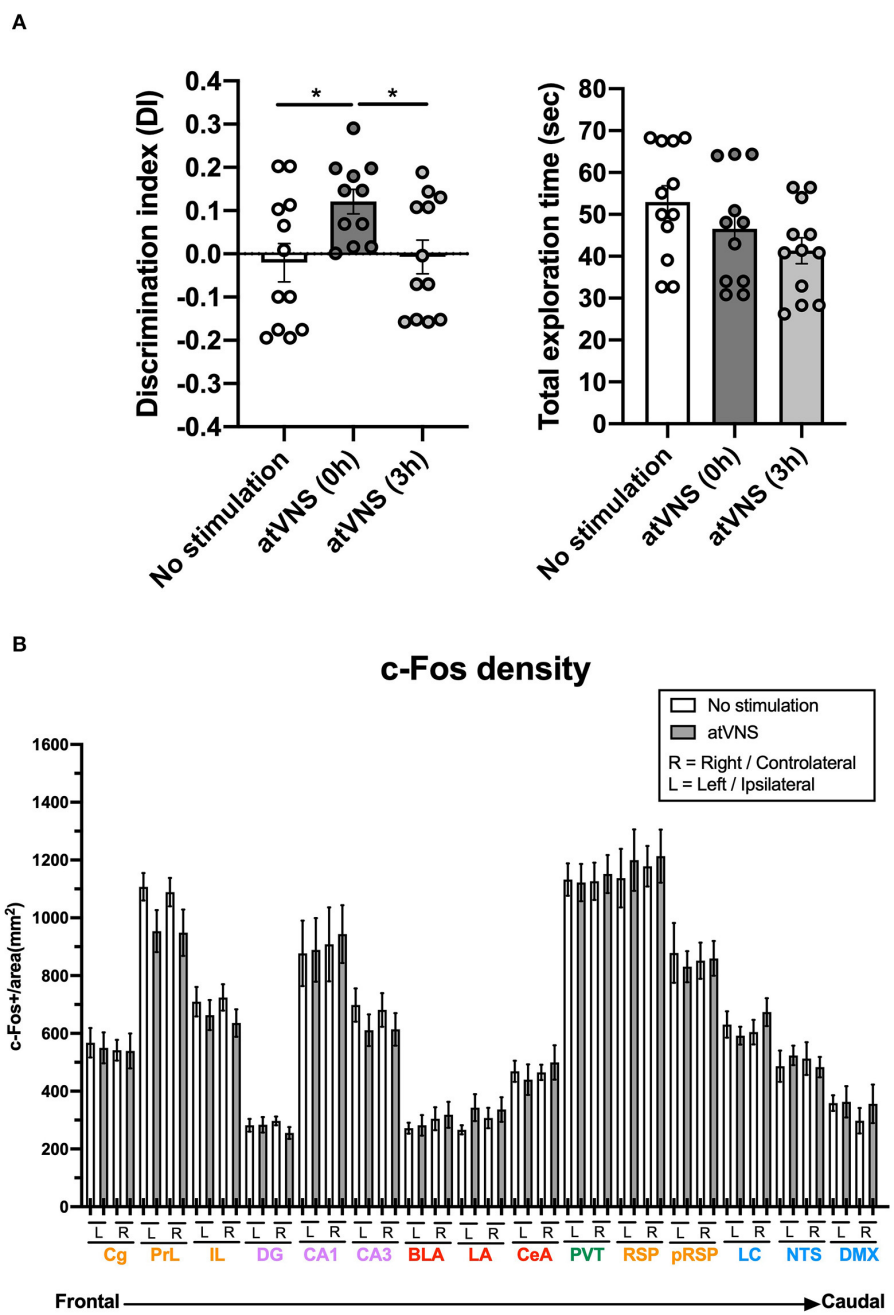
**(A)** atVNS improves object-recognition memory persistence in naïve mice when administered immediately after the familiarization phase of the novel object-recognition test (NORT). Discrimination index and total exploration time in NORT for atVNS (0 h), atVNS (3 h), and No stimulation conditions in naïve CD-1 mice [atVNS (0 h) condition, *n* = 11; atVNS (3 h) condition, *n* = 12; No stimulation condition, *n* = 12]. *p < 0.05 by the one-way ANOVA. **(B)** c-Fos density in No stimulation and atVNS (0 h) conditions, separating contralateral (right, R) and ipsilateral (left, L) sides according to the site of the stimulation. The brain regions analyzed are organized from frontal to caudal and grouped in the cortical (orange), hippocampal (purple), amygdalar (red), thalamic (green), and brainstem (blue) groups.

Auricular transcutaneous vagus nerve stimulation was administered after the familiarization phase of the NORT at two different time points, immediately after [atVNS (0 h) group] or 3 h after [atVNS (3 h) group]. As control, we intermingled another batch of animals that were similarly handled but did not receive the electrostimulation procedure (No stimulation group). We found that an acute session of atVNS, delivered immediately after the NORT familiarization phase, significantly improved object-recognition memory performance at 48 h (Figure 1A) compared with atVNS (3 h) and No stimulation conditions which showed similar results with no enhancement in memory persistence. This result indicates that the modulation of object-recognition memory persistence depends on the time of application after familiarization/training, with a critical time window for effective action of atVNS.

### c-Fos Density Is Not Significantly Modified in Various Brain Regions After atVNS

Brain samples from NORT + No stimulation and NORT + atVNS (0 h) [atVNS (0 h) was termed atVNS for the rest of the study] conditions were obtained 90 min after sham or atVNS handling to match the peak of expression of c-Fos. We focused on the analysis of c-Fos density in areas involved in novel object-recognition memory processing. Laterality was considered in the analysis.

Notably, c-Fos density analysis did not reveal significant differences between experimental conditions in any of the areas considered, although prelimbic and infralimbic regions and CA3 area showed a non-significant trend to reduce the density of c-Fos positive cells after atVNS (Figure 1B). This result showed that atVNS is not associated with localized regional changes, and therefore, we investigated whether its effects are related to a network reorganization of brain functioning.

### Inferred Brain Connectivity Is Relevantly Modulated by atVNS

**Figure 2:**
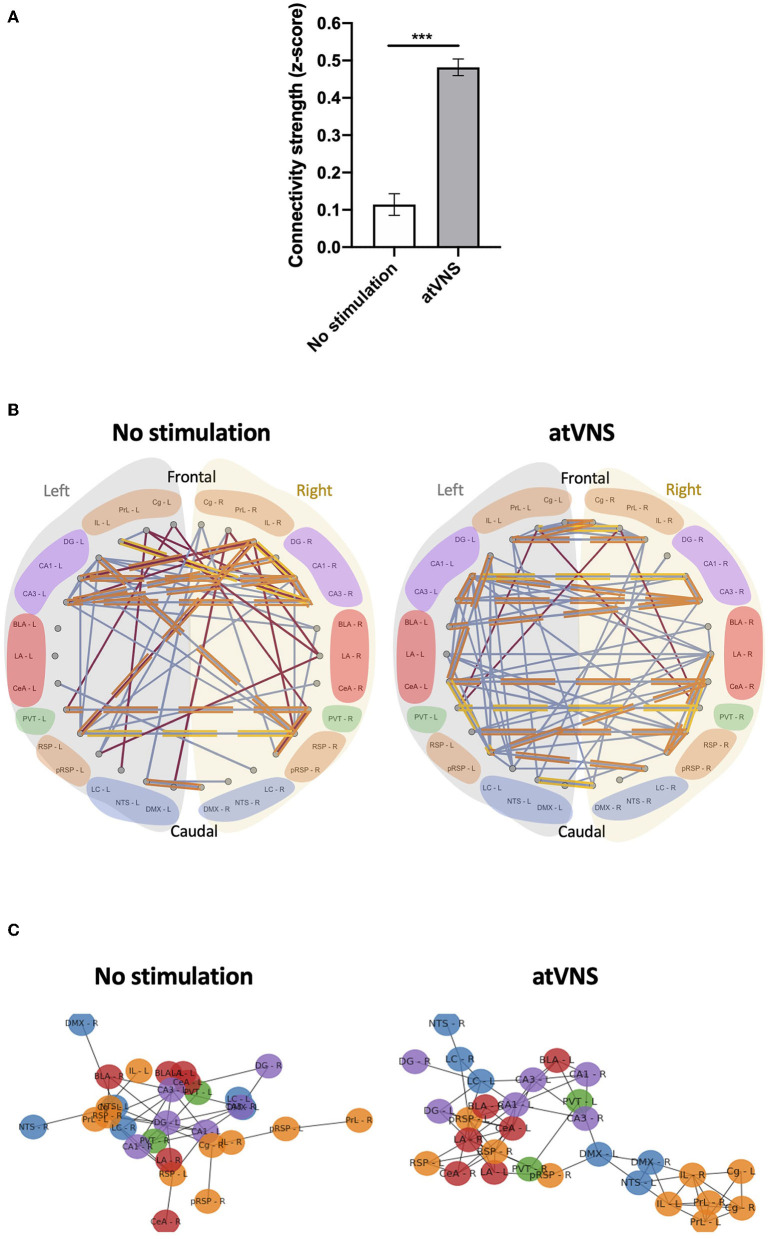
**(A)** Effects of atVNS on global network connectivity. Total connectivity presented as z-score, comparing No stimulation and atVNS (0 h) conditions. ***p < 0.001 by the Kruskal–Wallis test. **(B)** Network connectivity graphs displaying only the significant correlations (*p* < 0.05). Connecting lines represent Spearman correlation (positive correlation in blue, negative correlation in red). Strongest significant correlations are highlighted in orange (*p* < 0.01) and yellow (*p* < 0.001). Regions are presented from frontal to caudal and separating left (light gray) and right (light yellow) sides. Regions are grouped into the cortical (orange), hippocampal (purple), amygdalar (red), thalamic (green), and brainstem (blue) groups. **(C)** Network connectivity Kamada–Kawai plots displaying only positive significant z-score for No stimulation and atVNS (0 h) conditions. Colors represent the cortical (orange), hippocampal (purple), amygdalar (red), thalamic (green), and brainstem (blue) groups. Regions are grouped based on the connectivity strength between them.

To gain deeper insights into the functional connections within the set of brain regions in our analysis, we computed the connectivity matrices for each experimental group (Supplementary Figure 2). Comparing both connectivity matrices, an overall effect of atVNS on the relation of c-Fos density among different brain areas was observed, with a higher percentage of positive correlations under atVNS condition. To investigate deeper into this difference, we calculated the z-Fisher transform of correlations coefficients in both the conditions. The z-scores we obtained for each correlation coefficient represent the connectivity strength. When we statistically compared the strengths of all the connections between both conditions, we observed a significant increase in the total connectivity [No stimulation: 0.11 ± 0.029; atVNS: 0.48 ± 0.022; *t*_(868)_ = 10.05; *p* < 0.0001] induced by atVNS (Figure 2A).

Subsequently, we also focused on the networks created by significant correlations (*p* < 0.05). To disclose intra- and interhemispheric correlated activities for the network associated with each condition, we generated representative circos plots (Figure 2B). This analysis revealed that atVNS coupled the activity of left and right LC, also increasing its correlated activity with the dentate gyrus. Furthermore, the electrostimulation reinforced the relation in activity of all subregions of the hippocampus (DG, CA1, and CA3). Moreover, atVNS produced a marked enhancement in the correlated activity between hemispheres, especially in the frontal and amygdalar areas. A summary of intra- and interhemispheric significant correlations for each condition is shown in Table 1.

**Table 1:** Intrahemisphere and interhemisphere number of significant correlations in No stimulation and atVNS (0 h) conditions for frontal (Cg, PrL, and IL), hippocampal (DG, CA1, and CA3), amygdalar (BLA, LA, and CeA), and brainstem (LC, NTS, and DMX) areas.

**Areas**	**Intra-hemisphere**				**Inter-hemisphere**	
	**No stimulation**		**atVNS**		**No stimulation**	**atVNS**
	**Left**	**Right**	**Left**	**Right**	
Frontal cortex	3	5	4	5	7	10
Hippocampus	5	3	11	1	10	8
Amygdala	0	1	5	4	5	11
Brainstem	1	0	6	2	3	5

Notably, while there was an overall increase in connectivity after atVNS, the prefrontal-retrosplenial axis, characteristic of the default mode network, was not observed in control conditions, and atVNS did not have any marked effects on engaging this axis (Supplementary Figure 3).

In order to highlight the brain regions relevant in the network for each condition, we used the force-directed Kamada–Kawai plots. This representation revealed a re-organization of the network due to atVNS with a more evident cross-talk between the brainstem areas and frontal and hippocampal regions (Figure 2C). Indeed, atVNS produced a clear segregation of Cg, IL, and PrL cortices with left NTS and DMX as connection nodes to the remaining structures. Also, the amygdaloid nuclei and the RSP cortex took a central role that was occupied by the hippocampal nuclei under No stimulation conditions.

Global brain network metrics were used to account for the segregation and integration properties in each condition. We found a marked increase in all the evaluated properties in atVNS brains (global efficiency: No stimulation = 0.346, atVNS = 0.500; average clustering: No stimulation = 0.478, atVNS = 0.734; average strength: No stimulation = 4.037; atVNS = 5.956; average degree: No stimulation = 3.266; atVNS = 4.933).

**Figure 3:**
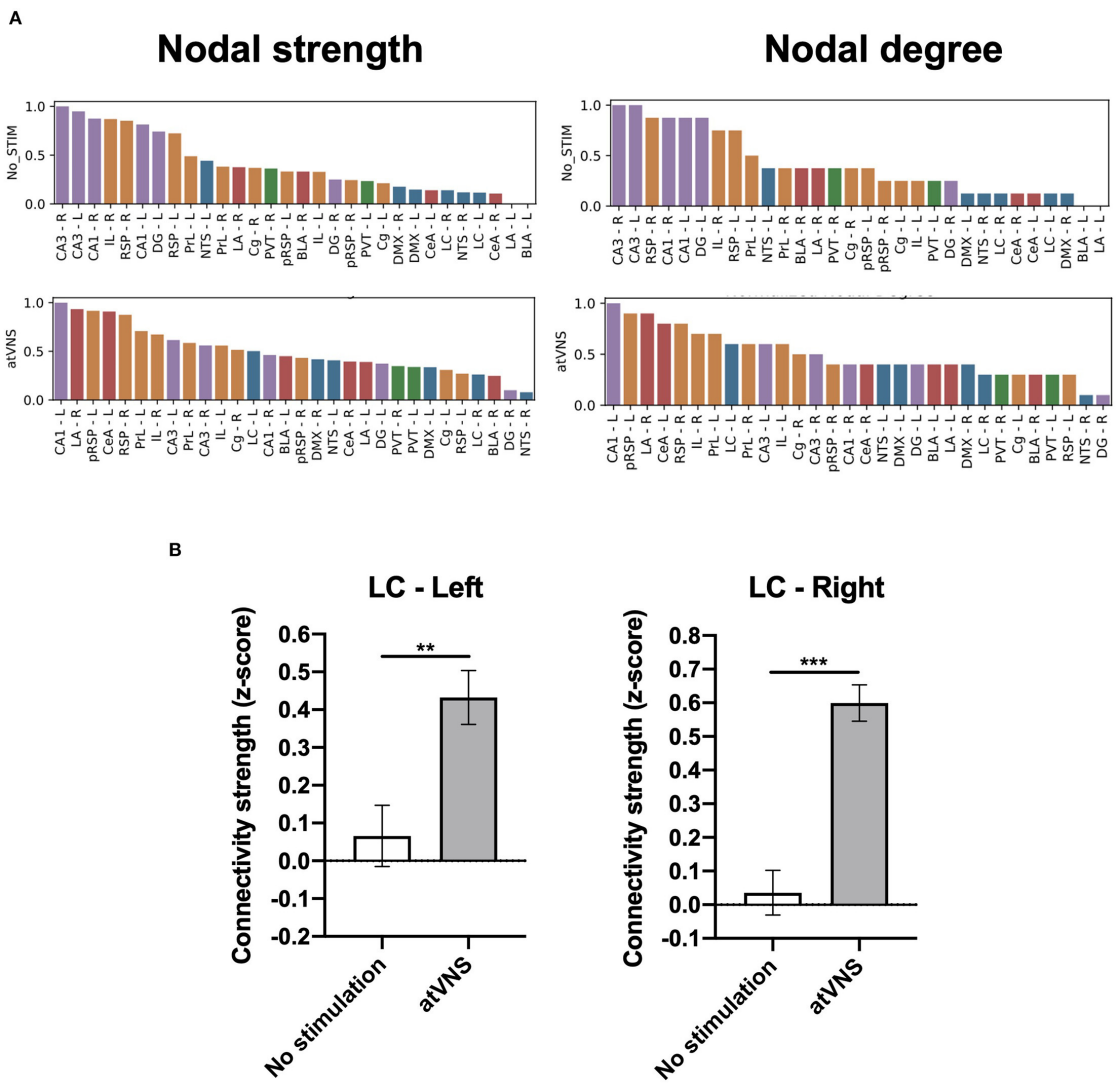
**(A)** Brain regions ranked in descending order based on nodal strength and nodal degree coefficients for No stimulation and atVNS (0 h) conditions. Regions are displayed with color coding: cortex (orange), hippocampus (purple), amygdala (red), thalamus (green), and brainstem (blue). **(B)** Difference in the total connectivity of right and left *locus coeruleus* (LC) regions with the rest of the brain areas analyzed, presented as z-score and comparing No stimulation and atVNS (0 h) conditions. **p < 0.01; ***p < 0.001 by the Kruskal–Wallis test.

We further assessed the relative importance of the brain regions analyzed in the overall brain network based on the regional network metrics. An increase in the relative importance of frontal areas in atVNS condition is observed, compared with No stimulation condition in which hippocampal regions showed a more relevant role. Furthermore, we found that brainstem regions, especially left LC, had a more relevant role in the atVNS network than in the No stimulation network, with a relatively higher value of both nodal strength and nodal degree coefficients (Figure 3A), since the brainstem regions constitute a passage between the vagus nerve afferents and superior brain regions. Indeed, if we compare the overall connectivity for the LC region, we observe a significant increase in the connectivity of both left and right LC (Figure 3B).

## Discussion

Invasive and non-invasive vagus nerve stimulations (VNSs) have been shown to modulate memory functions, both in animal (Clark et al., 1995, 1998; Vázquez Oliver et al., 2020) and human studies (Clark et al., 1999; Ghacibeh et al., 2006; Jacobs et al., 2015). However, the presence of an effective time window for atVNS effectiveness has not been explored before. In addition, the brain activity outcome of atVNS under memory retention facilitated conditions is still under study.

Therefore, first we aimed to determine whether there was a critical time window for effective enhancement of object-recognition memory persistence through an acute session of atVNS. We found that atVNS in the *concha* of the left external ear of naïve CD-1 male mice, improved memory persistence, when the electrostimulation protocol was delivered immediately after the familiarization phase of the NORT. Conversely, atVNS delivered 3 h after the training of the NORT did not show any sign of effect on recognition memory at 48 h. This evidence points out the presence of an effective time window for atVNS efficacy in modulating memory persistence. This result is in agreement with a previous clinical report describing that the stimulation, in this case through an invasive approach, was effective when given around the learning phase of a Hopkins Verbal Learning Test, and not during the recall phase (Ghacibeh et al., 2006). Therefore, both invasive and non-invasive forms of VNS, would principally enhance memory consolidation, leading to a better retention power when the electrical stimulus is delivered immediately after the familiarization or learning process.

Second, we investigated atVNS effects on neuronal and network activity, taking into consideration brainstem regions associated with vagal afferences, brain areas important for memory processes, and brain regions implicated in the DMN. It is well established that memory is not stored in a single brain area, but in a network composed of multiple regions (Tanimizu et al., 2017). In the case of recognition memory, the hippocampal formation comprises the main brain region involved (Brown and Aggleton, 2001), although cortical and subcortical areas are also engaged (Frankland and Bontempi, 2005). It has been postulated that memories are initially retained in the hippocampus, and then the information is transferred into the neocortex where it can be consolidated and stored for longer periods (Frankland and Bontempi, 2005; Insel and Takehara-Nischiuchi, 2013). Furthermore, the amygdala complex plays an important role in memory processes, especially under emotionally arousing experiences (Richter-Levin, 2004). These brain regions are contacted by brainstem regions which are relevant for setting the stage concerning the responsiveness associated with the arousal state (Roozendaal and McCaugh, 2011). In this regard, the LC can convey the information from vagal afferents arriving into the NTS (Grimonprez et al., 2015) and affecting the memory-related regions. Hence, we wondered whether the consolidation of new object-recognition memories, facilitated by atVNS, could be mediated by a change in the neuronal activation or a re-distribution of the activity relation between the brain areas.

Neuronal activation and functional connectivity were analyzed by computing the c-Fos density and interregional correlations across animals that received or not the electrostimulation procedure immediately after the familiarization phase of the NORT. We first bilaterally calculated the c-Fos density in a set of cortical, hippocampal, amygdalar, thalamic, and brainstem regions for No stimulation and atVNS conditions. Notably, atVNS did not promote significant changes in c-Fos density in any of the thirty (fifteen per hemisphere) brain regions studied. Other previous reports found changes in c-Fos density after the electrostimulation, particularly an increase in the number of c-Fos+ cells, in areas of the brainstem (Huffman et al., 2019; Jiang et al., 2019; Katagiri et al., 2019). However, all these studies used percutaneous or invasive VNS approaches, or applied the stimulation longer times. Our atVNS protocol instead is completely non-invasive, involves the auricular branch of the vagus nerve and the stimulation period is limited to 30 min, producing a significant effect in memory performance. Thus, the fact that no significant differences could be observed in the number of c-Fos+ cells in brain areas where other studies have found VNS-associated modulations, are probably due to the non-invasiveness and short stimulation protocol of atVNS procedure. Furthermore, several studies have been conducted using functional MRI (fMRI) and atVNS, especially in humans, to shed some light on the possible mechanisms and brain networks involved during atVNS. In general, left atVNS produced a significant activation in the ipsilateral NTS, LC and prefrontal and cingulate cortices, while bilateral deactivation was found in the hippocampus and hypothalamus, and controversial results were described in the amygdala (Kraus et al., 2007; Dietrich et al., 2008; Frangos et al., 2015; Butt et al., 2020). In contrast to fMRI procedure, we used c-Fos as a proxy for cellular activity; the main advantage of this approach is the cellular resolution mapping, although the poor temporal resolution is the principal limitation (McReynolds et al., 2018). Indeed, higher temporal resolution techniques would likely show distinct inter and intra-hemisphere differences that should be addressed in future studies. Additionally, all the animals were actively handled before sampling collections. This process could influence some of the results presented in the analysis, although extreme care was taken to make sure all animals were similarly handled, in order for the groups to only differ in the stimulation procedure. Notably, when we investigated c-Fos functional network, we found a significant reorganization, due to the electrostimulation procedure. atVNS resulted in an enhanced number of significant inter-hemisphere correlations compared to those observed in the No stimulation condition, especially in frontal and amygdalar areas. Moreover, significant correlations were found for sub-networks in the hippocampus (DG, CA1, and CA3) and frontal areas (PrL, IL, and Cg), with a specific increase in the correlation between the left LC and the dentate gyrus after atVNS. The distribution of the network differed between stimulation conditions. Under atVNS prefrontal cortices were segregated and linked to the remaining structures through DMX and left NTS, compared to No stimulation conditions, suggesting the ability of atVNS to dynamically reconfigure large-scale organization. In addition, a reorganization of the amygdala nuclei was observed pointing to a key role of this structure in the atVNS-mediated memory enhancement.

We also explored the effect of atVNS on selected DMN regions. This network has been also described in the mouse brain and involves the pRSP, RSP, Cg, PrL, and IL areas as components of the network (Stafford et al., 2014). By studying these areas in both hemispheres separately, we found that atVNS does not favor the connectivity of the DMN areas, as there is an absence of communication of the DMN anterior–posterior axis between frontal areas and retrosplenial cortex. This is in agreement with the idea that the DMN is mostly disengaged during task performance, and atVNS does not facilitate its engagement, and it may favor its disengagement.

Previous studies, using a combination of c-Fos expression and network analysis, found that long-term contextual fear memories are stored in a brain network composed by thalamic, hippocampal, and cortical regions (Wheeler et al., 2013), while the brain network composed by hippocampus, medial prefrontal cortex, anterior cingulate cortex, and amygdala was found required for the consolidation of social recognition memory (Tanimizu et al., 2017). These studies suggest that distinct types of memory are supported by exclusive functional memory networks that can be revealed by c-Fos analysis. Our findings in this study suggest that enhanced object-recognition memory consolidation is not prompted by an increase in neuronal activation by acute atVNS. Instead, we found a redistribution of the activity, and identified the correlation between brainstem nuclei and hippocampus and frontal areas as the privileged communication that may support the enhancement in memory persistence. Therefore, future studies should also focus on the real-time assessment of changes in neuronal activity induced by atVNS, to better understand its potential in modulating memory processes.

## Data Availability Statement

The raw data supporting the conclusions of this article will be made available by the authors, without undue reservation.

## Ethics Statement

The procedures involving experimentation on animals were reviewed and approved by the Barcelona Biomedical Research Park Experimentation Animal Ethical Committee and the local competent authorities.

## Author Contributions

CB-P participated in experimental design, conducted and analyzed experiments, and wrote the manuscript. EM-M produced network analysis and revised the manuscript. IG-A conducted and analyzed experiments. RM participated in the supervision and experimental design, funded the project, and revised the manuscript. AI participated in the supervision and stimulator design and generation, funded the project, and revised the manuscript. GS participated in the supervision and analysis of network data and revised the manuscript. AO conceptualized, participated in experimental design, supervised, funded the project, and wrote the manuscript. All authors reviewed and approved the final version of the manuscript.

## Funding

The project that gave rise to these results received the support of a fellowship from la Caixa Foundation (ID 100010434). The fellowship code is LCF/BQ/IN18/11660012 (CB-P). This project has received funding from the European Union's Horizon 2020 research and innovation program under the Marie Slodowska-Curie grant agreement No. 713673. This work was supported by the Ministerio de Economía, Innovación y Competitividad (MINECO) (#RTI2018-099282-B-I00 to AO and PID2020-120029GB-I00/MICIN/AEI/10.13039/501100011033 to RM); the Instituto de Salud Carlos III (#RD16/0017/0020 to RM and PI18/00893 cofunded by ERDF A way to make Europe to GS); the Generalitat de Catalunya (2017SGR-669 to RM); the ICREA (Institució Catalana de Recerca i Estudis Avançats) Academia to AO, AI, and RM; Grant Unidad de Excelencia María de Maeztu, funded by the MINECO (#MDM-2014-0370); and PLAN E (Plan Español para el Estímulo de la Economía y el Empleo). FEDER funding is also acknowledged.

## Conflict of Interest

The authors declare that the research was conducted in the absence of any commercial or financial relationships that could be construed as a potential conflict of interest.

## Publisher's Note

All claims expressed in this article are solely those of the authors and do not necessarily represent those of their affiliated organizations, or those of the publisher, the editors and the reviewers. Any product that may be evaluated in this article, or claim that may be made by its manufacturer, is not guaranteed or endorsed by the publisher.
