# Supplementary material for: Auricular Transcutaneous Vagus Nerve Stimulation Acutely Modulates Brain Connectivity in Mice
Source: Front Cell Neurosci. 2022 Apr 25;16:856855. doi: 10.3389/fncel.2022.856855 (PMC9081882; doi:10.3389/fncel.2022.856855)
Supplement: Supplementary file 1 [file Data_Sheet_1.pdf]

Supplementary Figures
Supplementary Figure 1.

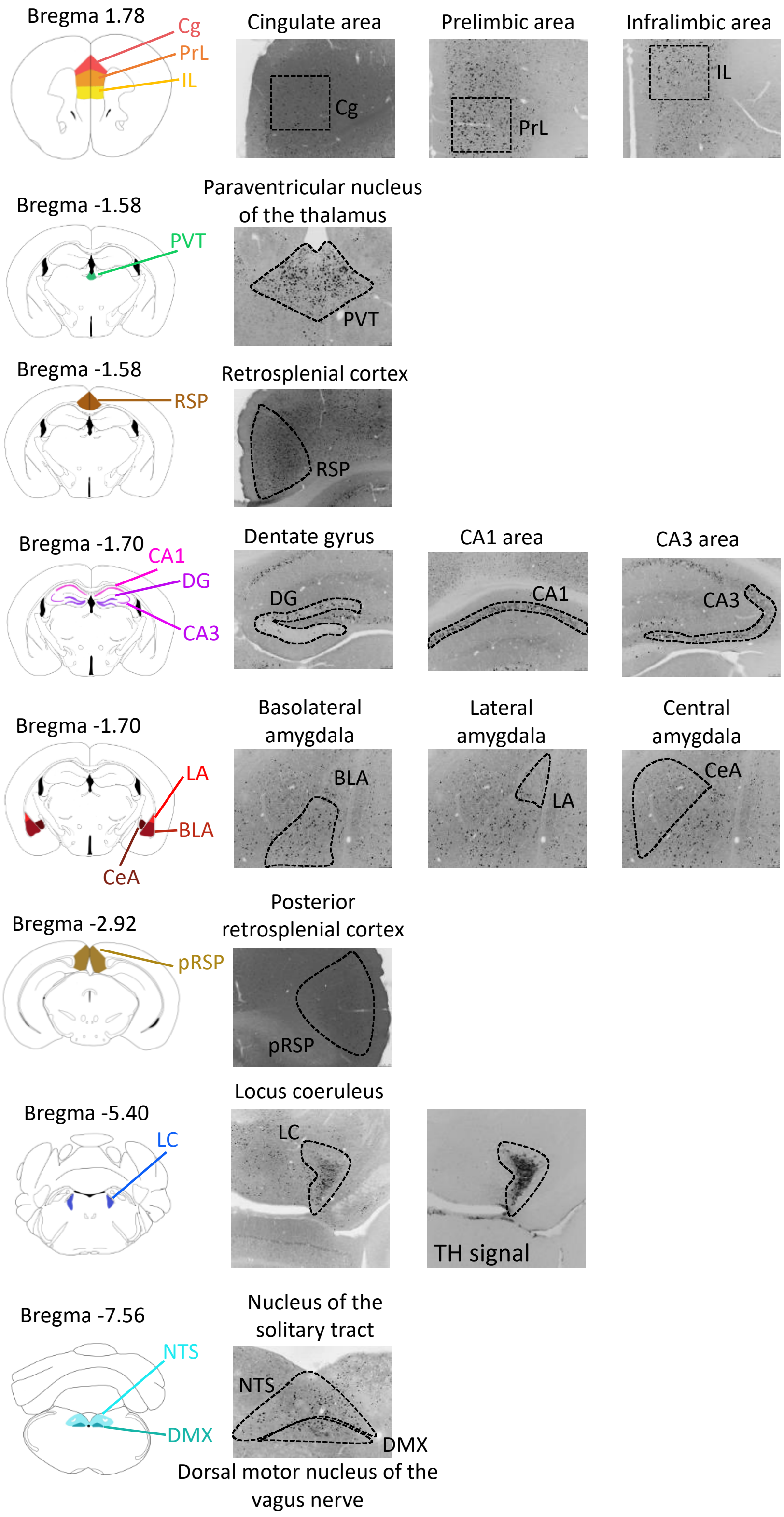

Brambilla-Pisoni et al., Supplementary Figure 1

Schematic representation and representative pictures of c-Fos immunofluorescence of brain region analyzed. Brain areas are displayed from frontal to caudal with the corresponding coordinates relative to Bregma.

Supplementary Figure 2.

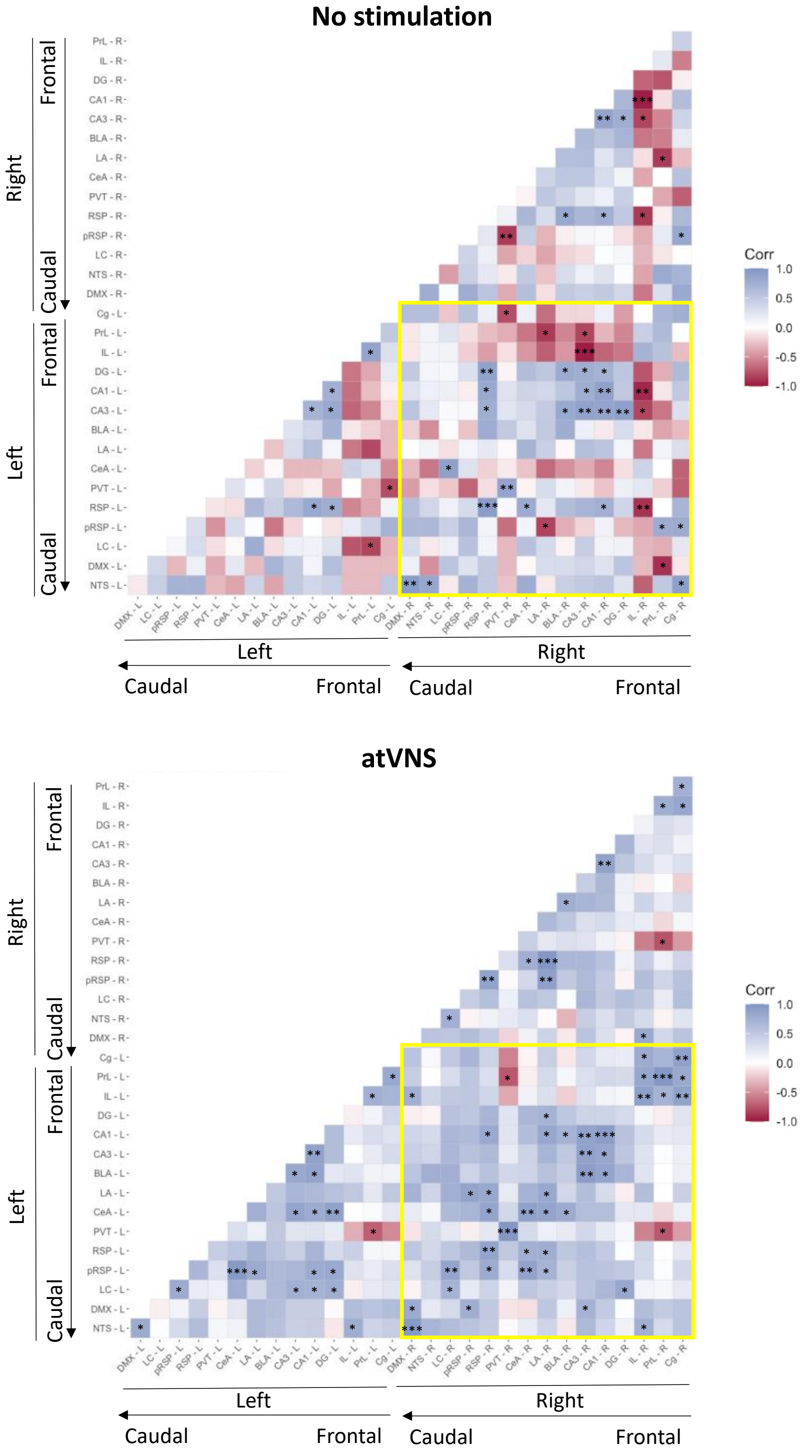

Brambilla-Pisoni et al., Supplementary Figure 2

Connectivity matrices showing inter-regional Spearman correlations for c-Fos density. Axes represent brain regions organized from frontal to caudal and separating left and right sides. The yellow square denote the interhemispheric connections. Colors reflect Spearman correlation coefficients (scale above), significant correlation is marked by * p<0.05, ** p<0.01 and *** p<0.001.

Supplementary Figure 3.

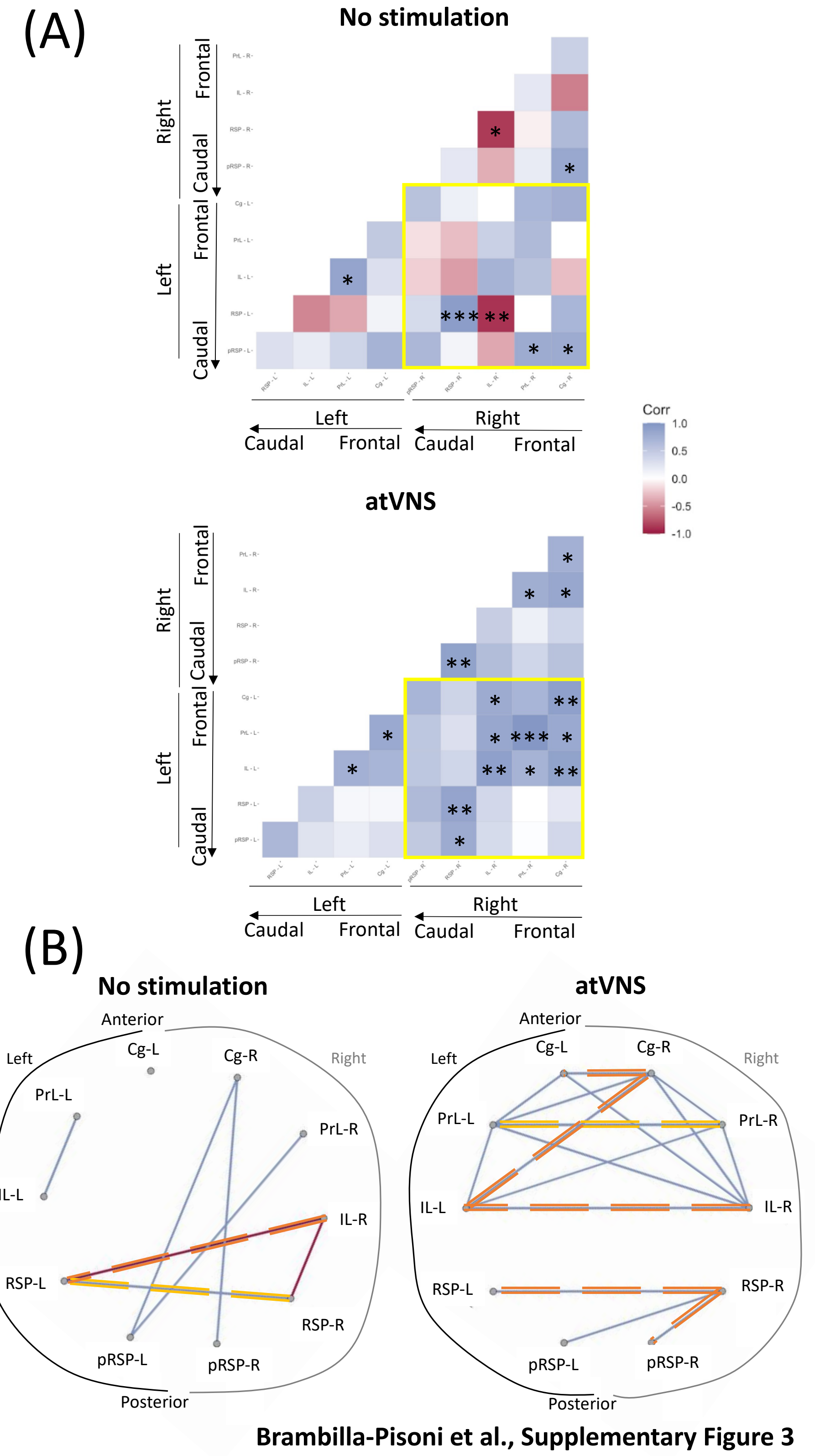

(A) Default mode network connectivity matrix. Axes represent brain regions organized from frontal to caudal and separating left and right sides. The yellow square denote the interhemispheric connections. Colors reflect Spearman correlation coefficients (scale above) and labels within squares correspond to p values of correlations (* p<0.05, ** p<0.01, *** p<0.001).

(B) Circle plots showing significant correlations (p<0.05) in default mode network areas. Connecting lines represent Spearman correlation (positive correlation in blue, negative correlation in red). Strongest significant correlations are highlighted in orange (p<0.01) and yellow (p<0.001). Regions are presented from frontal to caudal and separating left and right sides.
